# A Preliminary Study of Organ Weight After Histological Exclusion of Abnormality During Autopsy in the Adult Population of Uttarakhand, India

**DOI:** 10.7759/cureus.27044

**Published:** 2022-07-19

**Authors:** Vikas Vaibhav, Raviprakash Meshram, Pawan Kumar Shukla, Tushar Kalonia, Ashish R Bhute

**Affiliations:** 1 Forensic Medicine and Toxicology, All India Institute of Medical Sciences, Rishikesh, Rishikesh, IND; 2 Forensic Medicine and Toxicology, All India Institute of Medical Sciences, Raipur, Raipur, IND; 3 Pathology and Laboratory Medicine, All India Institute of Medical Sciences, Rishikesh, Rishikesh, IND

**Keywords:** lungs weight, brain weight, spleen weight, kidney weight, liver weight, heart weight, organomegaly, postmortem, autopsy, organ weight

## Abstract

Organomegaly can be a strong predictor of an underlying pathological condition. There are many standard tables available in various texts listing the normal organ weight range, yet there is a lack of a standard table that is accepted globally. The main reason behind this is variation in organ weight due to socioeconomic status, geographical variation, and racial and stature variation among different global populations. The Western population has different stature compared to our population, that is, residents of Uttarakhand, India.

Different studies tabulated organ weights in different regions of the world and correlated with different bodily parameters such as sex, race, stature, BMI, etc, which have shown a significant variation. There are different sets of data available that cannot be accepted universally due to regional variation. Most of the studies done in various parts of the world do not specify the condition of the organ, whether it was normal at the time of study or not. The methods of dissection of organs were also not explained in different studies.

In this study, a total of eight organs were weighed from 137 autopsies conducted at the mortuary of the All India Institute of Medical Sciences Rishikesh over a period of 1.5 years. It was found that the average brain weighed in males was 1313.2 gm (±127.7 gm) and among females, it was 1218.0 gm (±122.82 gm). The weight of the heart was 310.1 gm (±83.97 gm) in males and 241.2 gm (±71.42 gm) in females. Right and left lungs weighed 499.4 gm (±207.5 gm)/407.5 gm (±128.66 gm) and 459.6 gm (±179.19 gm)/369.4 gm (±144.17 gm) among males and females, respectively. The liver weight was 1477.0 gm (±370.52 gm) in males and 1309.0 gm (±274.18 gm) among females. Spleen weighed 154.0 gm (±74.63 gm) in males and 156.0 gm (±65.0 gm) in females. The right and left kidneys weighed 125.9 gm (±37.92 gm)/108.1 gm (±28.80 gm) and 126.3 gm (±31.26 gm)/106.6 gm (±22.4 gm) among males and females, respectively. In our study, we have done a histological examination to rule out any pathological condition before including the weight of the organs in the study.

The present study is to derive a standard organ weight among the inhabitants of Uttarakhand, India, and to look for a variation in organ weight among different studies done in the past in different regions of the world.

## Introduction

Organ weight can astoundingly reflect an underlying pathological condition. Some pathological conditions cause an increase in organ weight, whereas in some conditions there is shrinkage of organs, thereby a decrease in weight. Determining organ weight can give an important clue in differentiating a diseased organ from a normal organ, e.g., lungs weighing more than normal may point towards the possibility of pathology like heart failure (can be due to fluid accumulation) or pneumonia. On the other hand, reduced lung size guides us towards diseases like tuberculosis. Merely weighing an organ can guide a pathologist toward the root and the progression of a disease that could eventually play an important part in arriving at the cause of death.

The organ weight plays a significant role in forming the opinion regarding the cause of death in various pathological conditions and the use of organ weight at autopsy aids the forensic pathologist in the detection of gross anatomical abnormalities and pathology [1-4]. Organ weight can be a good diagnostic criterion during an autopsy if normality is accurately defined and known. Human organ weights besides race, age, gender, etc. are also anticipated to be dependent on environmental and socio-economic conditions, which may be quite different in various parts of the world. Hence, the organ weights reported from other parts of the world do not apply directly to the population elsewhere.

Histopathological examinations and their usefulness in post-mortem examination and medico-legal cases have continuously been thought to be very vital. Studies published in the past few years established the correlation between the weight of organs and body weight. Studies also show that male organ weight is more than female organ weight [5,6]. Some studies have been done to see the relationship between the weight of internal organs and body height, body weight, body mass index, and age [4,6]. A team of researchers from Colombia concluded that organ weight cannot be accurately predicted by body length, body weight, or body mass index [7]. Literature also suggests that all organ weights decline with the advancing age except the heart [8]. Studies also show that when the weight of the liver and brain were compared it was observed that except in children, the mean weight of the liver was more than the brain in both males and females [9].

There are some gross discrepancies between the observed weights of various organs at autopsy and that are given in standard textbooks [10]. A Korean study focused on the relationship between organ weight to body weight and concluded that the heart, spleen, and thyroid gland in males were not different from those in females, but the rest of the organs were heavier in males [11]. Organ weight decreases with age except for the thyroid and heart. Except for the heart, all the other organ weights showed a better statistical correlation with body length than BMI [12]. Post-mortem changes like decomposition and death due to any disease, fire, and drowning have significant effects on organ weight [10].

## Materials and methods

Study design

An observational study was conducted in the Department of Forensic Medicine and Toxicology at All India Institute of Medical Sciences, Rishikesh over 1.5 years after approval from the institutional ethics committee via letter no. AIIMS/IEC/18/541. A total of 335 autopsies were conducted during the period and 137 were included in the study after applying exclusion criteria. The weight of all the visceral organs was recorded at the time of autopsy and subsequently, sections were examined histologically. As weighing of organs and taking histology samples is the routine procedure during autopsy, no additional consent was taken.

The sample included eight organs - brain, heart, liver, kidneys, both lungs, and spleen each from 137 autopsies - which were weighed and histological examination was done for any alteration. Cases included were residents of Uttarakhand by domicile, aged between 15-55 years, among the bodies subjected to a medico-legal autopsy, and having time since death within 18 hours (prolonged time since death may alter the organ weight) [13]. Case excluded were any diseased organ having any histological alteration, decomposed bodies, injured organs, any anatomically abnormal organ, cases of poisoning, burn, drowning and snake bite, and unidentified bodies.

Organs obtained from the autopsy were weighed on a digital electronic weighing scale which was calibrated daily prior to each autopsy. An autopsy was conducted as per the standard protocol and procedure described in *Current Methods of Autopsy Practices* by J. Ludvig [14]. All organs included in the study were removed by Ghon’s Method, in which cervicothoracic, abdominal, and pelvic organs are removed as three separate blocks (en bloc method). Before weighing, all extraneous tissues and blood clots were removed.

The heart was separated at the root of the aorta, dissected along the line of blood flow, blood clots removed, and the dissected heart was washed before weighing. The lungs were removed from the thoracic en bloc at the hilum in both the lungs and the mucus plug if any was removed before weighing. The brain was cut at the level of the spinal cord then part of the cord was separated; the brain was weighed along with the brain stem. The liver was dissected and separated from the diaphragm and vessels were cut, ligaments removed, and the gall bladder was removed before weighing. Spleen was separated from all vessel attachments and removed. Kidneys were cut at hilum vessels and ureter and para-renal fat was removed before weighing.

**Figure 1:**
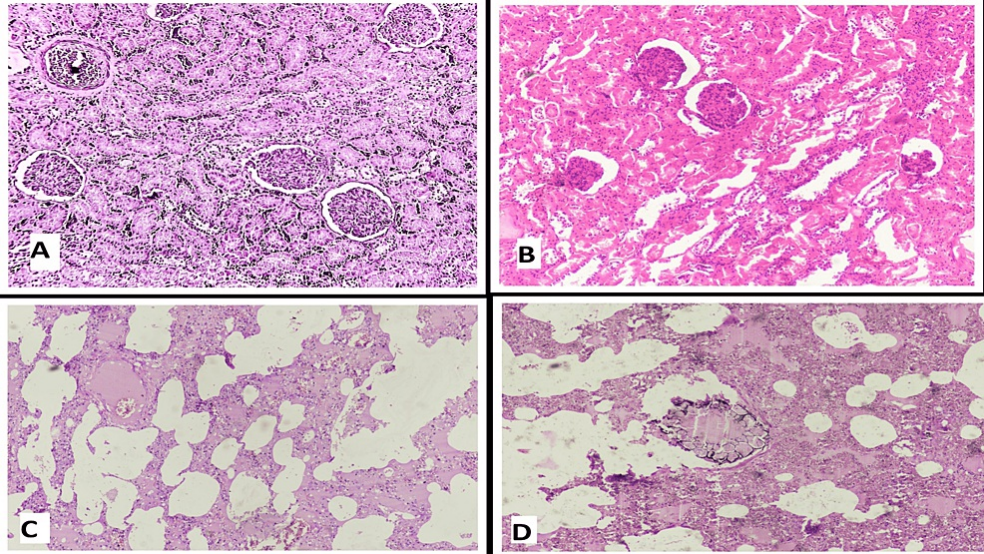
A - Congested kidney, B -Sclerosed glomeruli having patchy thyroidisation and tubular necrosis with inflammation, C - Marked pulmonary edema, D - Lung with a foreign body Haematoxylin & Eosin stain, 10x

**Figure 2:**
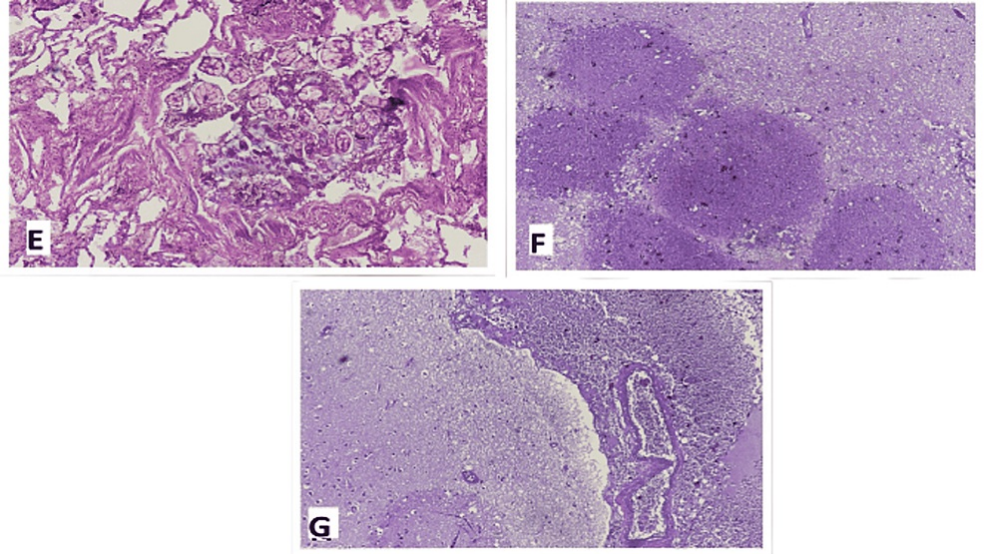
E - Lungs showing aspiration pneumonitis, F - Brain showing infarcted area, G - Brain showing subarachnoid hemorrhage Haematoxylin & Eosin stain, 10x

If any visible abnormality in any organ were present, sections were taken from that part, otherwise, numerous sections were taken from different parts of organs to rule out any invisible histological abnormality. For histological analysis, all organs removed from the body were grossed and sections were taken, preferably one section from each organ, which was then subjected to processing with the help of an automated tissue processor using various chemicals like formalin, alcohol, paraffin, and xylene. A processed tissue was then embedded and paraffin blocks were made in an embedding station. Four microns (µm) sections were cut from blocks and subjected to automated Haematoxylin & Eosin stainer for staining. Stained slides were mounted by a suitable medium & were observed under a microscope for any histological alteration (Figure 1A-1D, Figure 2E-2G).

## Results

The results were calculated using 'R' statistical software version R4.2.0 (R Foundation, Vienna), for calculating the Mean and Standard Deviation (SD). The organ weight relationship according to gender was correlated statistically using the t-test. The mean is the average where we add up all the numbers and then divide by the total variables. Standard Deviation is a statistical parameter that measures the dispersion of the dataset relative to its mean and is calculated as the square root of the variance. It is calculated as the square root of variance by determining the variation between each data point relative to the mean. The t-test is a statistical tool for comparing the mean values of two sets of numbers. The comparison provides a statistic for evaluating whether the difference between two means is statistically significant. It can be used to compare two independent groups (independent sample t-test) or compare observations from two measurement occasions from the same group (paired sample t-test).

**Figure 3:**
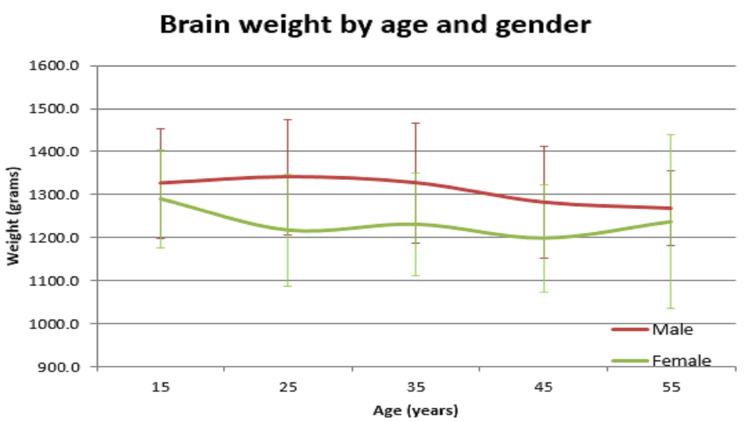
Brain vs age in both males and females

**Figure 4:**
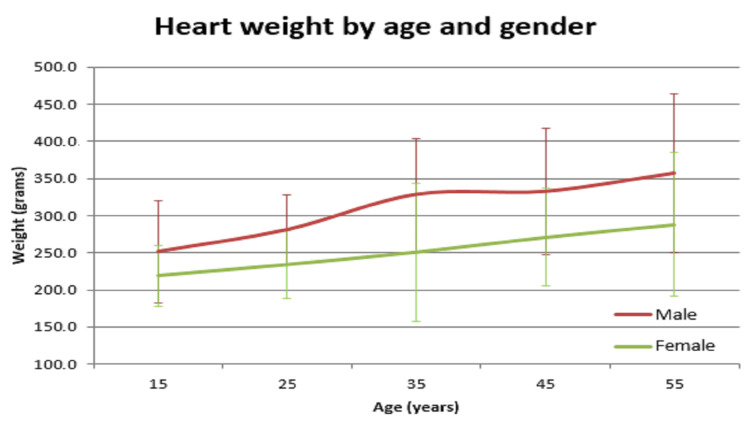
Heart vs age in male and female

**Figure 5:**
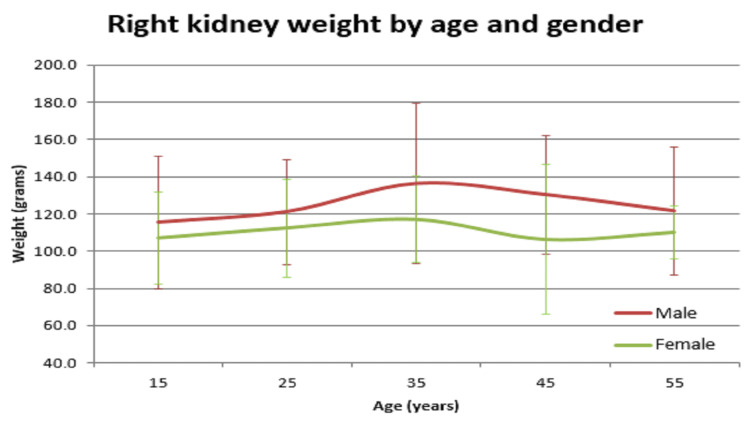
Right kidney vs age in both male and female

**Figure 6:**
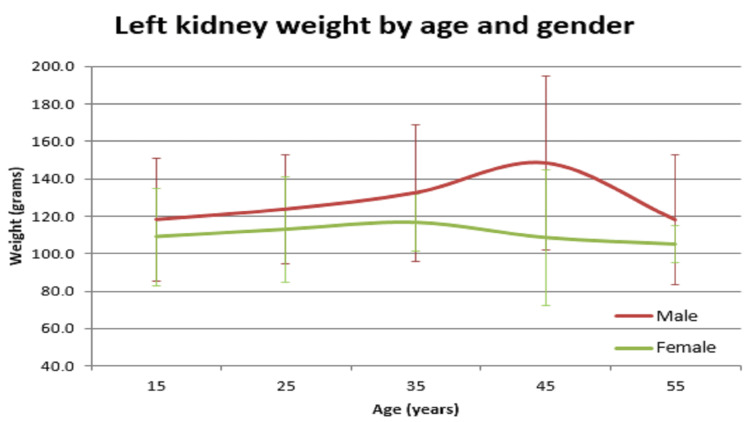
Left kidney vs age in both males and females

**Figure 7:**
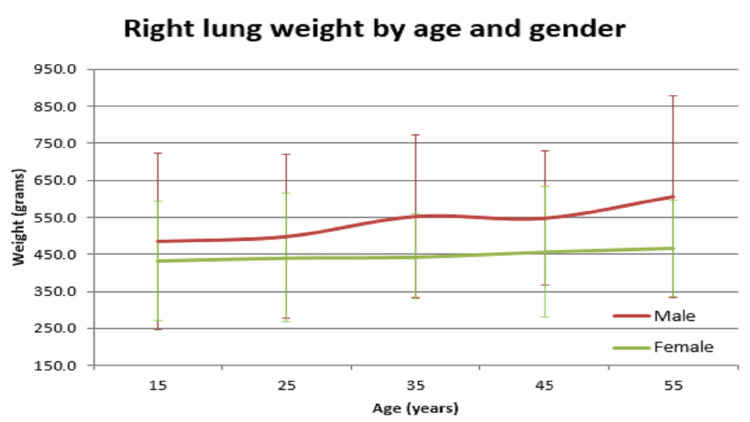
Right lung vs age in both males and females

**Figure 8:**
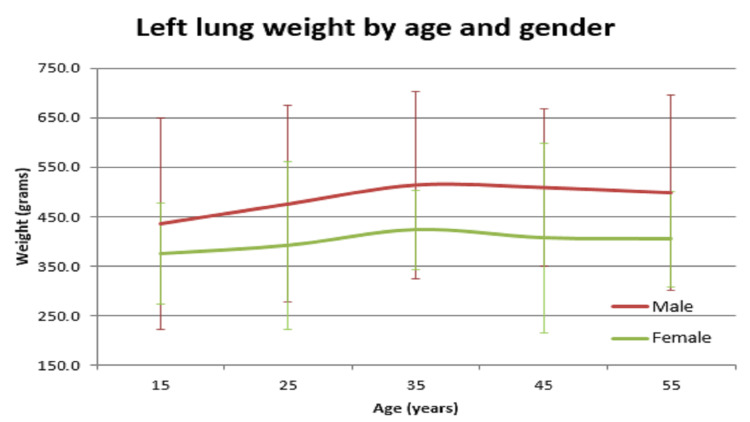
Left lung vs age in both male and female

**Figure 9:**
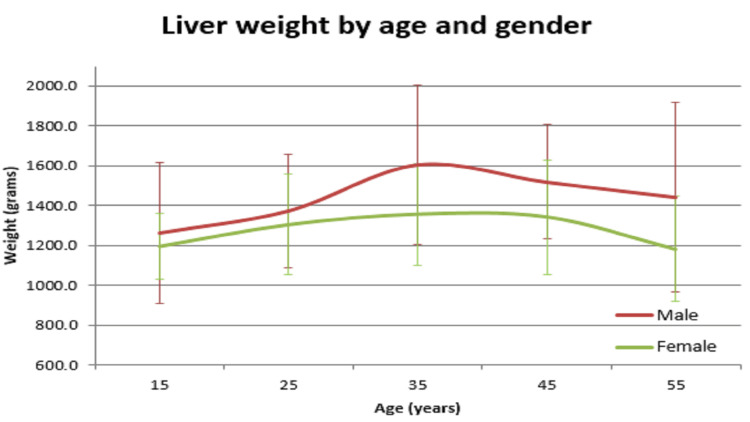
Liver vs age in both males and females

**Figure 10:**
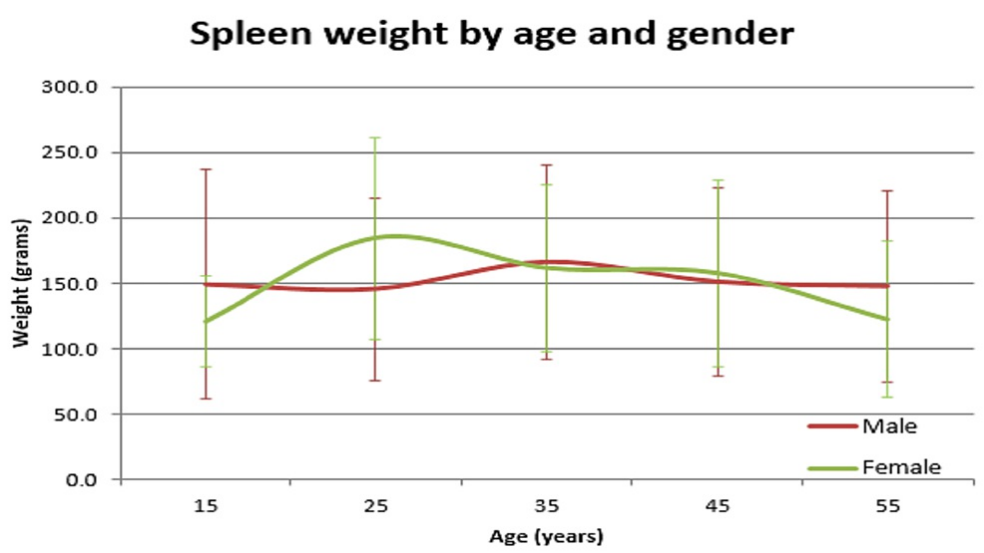
Spleen vs age in male and female

In our study, the average normal organ weight in grams among both sexes (male/female) was: brain weighed 1313.2 gm ± 127.7 gm in males and 1218.0 gm ± 122.82 gm in females, heart weighed 310.1 gm ± 83.97 gm and 241.2 gm ± 71.42 gm in females, right lung weighed 499.4 gm ± 207.5 gm in males and 407.5 gm ± 128.66 gm in females, left lung weighed 459.6 gm ± 179.19 gm in males and 369.4 gm ± 144.17 gm in females, liver weighed 1477.0 gm ± 370.52 gm in males and 1309.0 gm ± 274.18 gm in females, spleen weighed 154.0 gm ±74.63 gm in males and 156.0 gm ± 65.0 gm in females, right kidney weighed 125.9 gm ± 37.92 gm in males and 108.1 gm ± 28.80 gm in females, left kidney weighed 126.3 gm ± 31.26 gm in males and 106.6 gm ± 22.4 gm in females (Table 1). The mean of all organ weights was higher in males than in females except in the case of the spleen. The t-test was applied and it was found that all organs showed statistically significant results, except the spleen, which was not statistically significant (Table 2). Organ weight among different age groups in males and females was as given in Table 3 and Table 4. There was a variation in organ weight among the males and females as shown in Figures 3-10. 

**Table 1:** Average normal organ weight in grams among both sexes (male/female) n = 137; Rt = right; Lt = left; SD = Standard Deviation

Variables	Male (Mean ± SD, n=104)	Female (Mean ± SD, n=33)
Brain	1313.2 ± 127.7	1218.0 ± 122.82
Heart	310.1 ± 83.97	241.2 ± 71.42
Rt Lung	499.4 ± 207.5	407.5 ± 128.66
Lt Lung	459.6 ± 179.19	369.4 ± 144.17
Liver	1477.0 ± 370.52	1309.0 ± 274.18
Spleen	154.0 ± 74.63	156.0 ± 65.0
Rt Kidney	125.9 ± 37.92	108.1 ± 28.80
Lt Kidney	126.3 ± 31.26	106.6 ± 22.4

**Table 2:** Correlation of organ weight in grams between males and females with the p-value Rt = right; Lt = left; ⁕ = statistically significant

Organ	Sex		Independent Samples t-test (p-value)
	Male	Female	
Brain	1313.2	1218.0	< 0.001⁕
Heart	310.1	241.2	< 0.001⁕
Rt Lung	499.4	407.5	0.003⁕
Lt Lung	459.6	369.4	0.005⁕
Liver	1477.0	1309.0	0.008⁕
Spleen	154.0	156.0	0.8
Rt Kidney	125.9	108.1	0.007⁕
Lt Kidney	126.3	106.6	< 0.001⁕

**Table 3:** Organ weight in grams in males among different age groups Rt = right; Lt = left; Yrs = years

Age(Yrs)	Subjects	Variable	Brain	Rt Lung	Lt Lung	Heart	Liver	Spleen	Rt Kidney	Lt Kidney
<25	24	Mean	1322	453.8	410.6	274.5	1308	147.9	118.3	115.0
		SD	129.8	196.5	183.5	57.5	247.087	87.2	26.0	19.1
		Range	1050-1590	220-1110	190-1040	190- 420	900-1920	70-490	80-190	80-170
25-34	34	Mean	1339	503	469.8	282.6	1439	147.9	127.4	126.6
		SD	107	249.8	208	49.7	302.1	69.7	47.9	34.1
		Range	1530-1110	120-1270	120-1080	190-380	1070-2220	60- 320	80-350	70-190
35-44	37	Mean	1300	526	477.2	349.2	1641	164.9	131.6	132
		SD	150.8	177.8	156.5	98.7	456.2	74.1	35.9	34.9
		Range	1050-1820	210-920	200-860	170-630	860-2990	40-300	60-230	60-220
45-55	9	Mean	1313	504.4	482.8	344.4	1391	151.1	116.7	132.2
		SD	105.9	184.5	136.9	107.6	232.9	65.8	30	25.9
		Range	1190-1530	320-820	260-670	250-580	1020-1710	50-240	80-160	90-160

**Table 4:** Organ weight in grams in females among different age groups Rt = right; Lt = left; Yrs = years

Age(Yrs)	Subjects	Variable	Brain	Rt Lung	Lt Lung	Heart	Liver	Spleen	Rt Kidney	Lt Kidney
<25	10	Mean	1270	368	325	223	1334	152.2	111.1	104.4
		SD	102.5	103.8	93.7	41.6	159.1	47.4	19.6	20.1
		Range	1150-1480	140-480	130-460	160-280	980-1560	90-230	90-140	80-130
25-34	10	Mean	1204	402	358	242	1277	163.3	106	105
		SD	110.6	113.5	143.4	89.5	337.7	74.6	30.6	24.6
		Range	1010-1400	170-570	100-560	150-440	800-1880	90-320	60-150	70-150
35-45	9	Mean	1176	445	415.8	255.8	1313	155.5	107.5	109.6
		SD	141.7	115.9	174.1	76.6	308.9	74.5	34.7	23.8
		Range	960-1380	160-650	150-730	160-450	820-1920	80-320	30-160	70-150

## Discussion

Different studies across the country showed similar findings as seen in our study. According to Prakash et al, all organ weights were higher in males [1]. Similar results were obtained by Bhoi SB et al, Singh et al, Puttaswamy, Kim et al, and Narongchai et al [5, 8, 9, 11, 15]. Some studies also showed that dark-skin descendants have heavier organ weights than their white counterparts [16]. Western populations have heavier organ weights than the study population [1].

In our study when the correlation of organ weight was studied between males and females, it showed that, the brain (p<0.001), heart (p<0.001), right lung (p=0.003), left lungs (p=0.005), liver (p=0.008), right kidney (p=0.007), and left kidney (p<0.001) were showing statistically significant results when the t-test was applied, except for spleen (p=0.8), which was not statistically significant (Table 2).

Similar findings were noted by Prakash et al; they found that the weight of the spleen was higher in females [1]. Another study by Bhoi et al showed that the weight of the spleen was higher in males [5]. Sprogøe-Jakobsen et al showed there was no difference in spleen weight among both sexes [17]. Similarly, a study among the Bengali population by Bandyopadhyay and Biswas showed that the mean weight of the spleen in males was higher than that of females [3]. A study done at a South African mortuary showed that there was no correlation of organ weight between the sexes. Only heart (p=0.013), right lung (p<0.001), left lung (p<0.001), and liver (p=0.006) showed statistically significant values. Other organs - spleen (p=0.305), right kidney (p=0.210), and left kidney (p=0.200) - were statistically insignificant. There was a significant variation in organ weight seen among both the genders among different age groups (Tables 3,4 and Figures 3-10).

One of the main limitations of the study is the number of subjects included in the study was less; to draw a more strong and more reliable guideline for normal organ weight, the number of subjects included should be increased and studies should be conducted over a longer period for more consistent results.

## Conclusions

The organ size and weight is a complex interaction of biological and ecological constraints. Mechanisms involved in the regulation of organ size must be synchronized correctly for the survival and proper functioning of the organ. Organ weight has a major influence on a forensic pathologist while drawing an inference during a medico-legal autopsy. The use of incorrect and inappropriate standard values can result in inaccurate results. In our study conducted among people of Uttarakhand, we found that the internal organs in males were heavier than those in females. Internal organ weight also tends to increase with increasing age. In forensic medicine use of incorrect data sets for internal organs weight may lead us to wrong judgments, most of them as histological examinations are not a routine practice. So there must be an updated reference table of normal internal organ weight after proper exclusion of abnormality. In our study, it was noted that male organs were heavier than that of females as all organs significantly correlated with gender except in the case of the spleen which was statistically not significant. All organ weights increased with advancing age. Significant abnormalities were ruled out while carrying out histological analysis in assigning any organ normal. The results from studies done among the Western population showed heavier organ weight than our results; this means that the weights that are considered normal in the Western population could be abnormal in our demography. Newer modalities like postmortem computed tomography (PMCT) can also be used to calculate the organ weight without dissecting the bodies.
